# Linking Oxidative Events to Inflammatory and Adaptive Gene Expression Induced by Exposure to an Organic Particulate Matter Component

**DOI:** 10.1289/ehp.1104055

**Published:** 2011-10-13

**Authors:** Wan-Yun Cheng, Jenna Currier, Philip A. Bromberg, Robert Silbajoris, Steven O. Simmons, James M. Samet

**Affiliations:** 1 Department of Environmental Sciences and Engineering, Gillings School of Global Public Health,; 2 Curriculum in Toxicology, UNC School of Medicine, and; 3 Center for Environmental Medicine, Asthma and Lung Biology, UNC School of Medicine, University of North Carolina at Chapel Hill, Chapel Hill, North Carolina, USA; 4 Environmental Public Health Division, National Health and Environmental Effects Research Laboratory, U.S. Environmental Protection Agency, Chapel Hill, North Carolina, USA; 5 Integrated Systems Toxicology, National Health and Environmental Effects Research Laboratory, U.S. Environmental Protection Agency, Research Triangle Park, North Carolina, USA

**Keywords:** confocal microscopy, hydrogen peroxide, mitochondrial dysfunction, oxidative stress, quinones, reactive oxygen species, real-time imaging, ROS

## Abstract

Background: Toxicological studies have correlated inflammatory effects of diesel exhaust particles (DEP) with its organic constituents, such as the organic electrophile 1,2-naphthoquinone (1,2-NQ).

Objective: To elucidate the mechanisms involved in 1,2-NQ–induced inflammatory responses, we examined the role of oxidant stress in 1,2-NQ–induced expression of inflammatory and adaptive genes in a human airway epithelial cell line.

Methods: We measured cytosolic redox status and hydrogen peroxide (H_2_O_2_) in living cells using the genetically encoded green fluorescent protein (GFP)-based fluorescent indicators roGFP2 and HyPer, respectively. Expression of interleukin-8 (*IL-8*), cyclooxygenase-2 (*COX-2*), and heme oxygenase-1 (*HO-1*) mRNA was measured in BEAS-2B cells exposed to 1,2-NQ for 1–4 hr. Catalase overexpression and metabolic inhibitors were used to determine the role of redox changes and H_2_O_2_ in 1,2-NQ–induced gene expression.

Results: Cells expressing roGFP2 and HyPer showed a rapid loss of redox potential and an increase in H_2_O_2_ of mitochondrial origin following exposure to 1,2-NQ. Overexpression of catalase diminished the H_2_O_2_-dependent signal but not the 1,2-NQ–induced loss of reducing potential. Catalase overexpression and inhibitors of mitochondrial respiration diminished elevations in *IL-8* and *COX-2* induced by exposure to 1,2-NQ, but potentiated *HO-1* mRNA levels in BEAS cells.

Conclusion: These data show that 1,2-NQ exposure induces mitochondrial production of H_2_O_2_ that mediates the expression of inflammatory genes, but not the concurrent loss of reducing redox potential in BEAS cells. 1,2-NQ exposure also causes marked expression of *HO-1* that appears to be enhanced by suppression of H_2_O_2_. These findings shed light into the oxidant-dependent events that underlie cellular responses to environmental electrophiles.

Oxidant stress is a commonly described mechanistic feature of the toxicity of environmental contaminants (Monks et al. 1992). Multiple pathophysiological effects of environmental exposures, including cancer, fibrosis, and inflammation, have been associated with oxidant damage to macromolecules such as lipids, proteins, and DNA (Kelly et al. 1998). Oxidant stress induced by a toxicant is invariably a multifaceted process involving exogenous and endogenous reactions between xenobiotic and cellular macromolecules. Toxic exposures often elicit cellular responses that are intrinsically oxidant in that they involve production of reactive oxygen species (ROS) and/or the loss of intracellular reducing potential. Oxidative cellular responses to exposure to oxidizing agents can also occur, and thus the elucidation of the events involved and the order in which they occur presents significant analytical challenges (Ercal et al. 2001; Santa-Maria et al. 2005; Steinberg et al. 1990; Valko et al. 2005).

Oxidant stress is believed to play an important role in air pollutant–mediated toxicity in the respiratory tract. Transition metals and organic chemical components of diesel exhaust particles (DEP) have been shown to induce the generation of various ROS (Monks et al. 1992), including the superoxide radical, hydrogen peroxide (H_2_O_2_), and nitric oxide (Kumagai et al. 1997; Li et al. 2003). The relationship between oxidative stress and altered expression of inflammatory and adaptive genes has been well established for a variety of air pollutants (Becker et al. 2005; Rahman and MacNee 2000).

Although established methods for the measurement of oxidant damage to cells and tissues exist, they are relatively insensitive and often provide only inferential mechanistic information. In contrast, detection of primary oxidative events resulting from environmental exposures is inherently challenging because of the transient nature of the events involved, as well as the relatively low levels of oxidant reactants that are generated. Imaging approaches offer the distinct advantages of providing high temporal and spatial resolution, as well as the high sensitivity necessary to detect early indicators of oxidative stress in cells exposed to environmental agents. Recently, we described an integrated imaging approach for the real-time measurement of redox potential changes and H_2_O_2_ generation resulting from mitochondrial dysfunction in living cells exposed to the nonredox-active transition metal Zn^2+^ (Cheng et al. 2010). In the present study, we expanded this approach to include an investigation of the relationship between specific oxidant events in the cytosol and mitochondria and altered gene expression induced by the redox-active air contaminant, 1,2-naphthoquinone (1,2-NQ).

1,2-NQ, a reactive electrophile associated with diesel exhaust particles (DEP) (Bai et al. 2001; Rodriguez et al. 2004), has been shown to have cytotoxic, mutagenic, and immunotoxic effects (Monks et al. 1992). Quinone toxicity has been found to involve two primary initiating mechanisms: *a*) a 1,4-Michael addition reaction leading to covalent modification of cellular targets (Endo et al. 2007; Miura et al. 2011) and *b*) ROS generation through redox cycling (Rodriguez et al. 2004). Previous studies have shown that 1,2-NQ attacks protein–tyrosine phosphatases (Iwamoto et al. 2007; Kikuno et al. 2006; Sun et al. 2006), which has been associated with the activation of signaling pathways that can lead to the expression of proinflammatory proteins such as interleukin-8 (IL-8) and cyclooxygenase-2 (COX-2) (Kuwahara et al. 2006; Tsatsanis et al. 2006) and the adaptive protein HO-1 (Kuroda et al. 2010). Multiple studies have suggested a role for ROS generation and inflammatory processes, but the link between oxidant stress and inflammatory and adaptive gene expression has not been examined following exposure to environmental electrophiles.

Here we report that exposure to 1,2-NQ results in a rapid loss of intracellular reducing potential and increased production of H_2_O_2_ of mitochondrial origin, and that these end points associate differentially with the induction of inflammatory and adaptive gene expression.

## Materials and Methods

*Reagents.* Tissue culture media and supplements were obtained from Lonza (Walkersville, MD). Adenoviral vectors were procured from the Gene Therapy Center Virus Vector Core Facility (University of North Carolina at Chapel Hill). Common laboratory reagents were obtained from Sigma Chemical Co. (St. Louis, MO). Basic laboratory supplies were purchased from Fisher Scientific (Raleigh, NC).

*Synthesis of fluorescent reporter genes in lentiviral vector.* The genetically encoded fluorescent reporter roGFP2 is a redox-sensitive ratiometric probe established for detection of oxidative stress in the cytosol and mitochondria (Hanson et al. 2004). The plasmid for this protein was a generous gift from S.J. Remington (University of Oregon, Eugene, OR). HyPer is a genetically encoded probe specific for H_2_O_2_ detection and was purchased from Evrogen (Axxora, San Diego, CA). The two genes, *roGFP2* and *HyPer*, were isolated from pEGFP-N1 and pQE30 vector by *BamH*I and *Hind*III digest and cloned into the lentiviral transfer vector pTLRED [U.S. Environmental Protection Agency (EPA)]. HEK293T cells were cotransfected with purified transfer vector plasmids and lentiviral packing mix (Open Biosystems, Huntsville, AL). The resulting supernatants from the individual transfections were concentrated once by low-speed centrifugation through an Amicon Ultra 100kD centrifuge filter unit (Millipore, Billerica, MA), and the retentates were aliquoted and stored at –80°C. Viral titers were determined in HEK293T cells stably expressing the rTTA3 transactivator (E10 cells) by transduction with serially diluted vector stocks as previously described (Simmons et al. 2011).

*Cell culture and viral transduction.* Transformed human airway epithelial cells (BEAS-2B) (Reddel et al. 1988) were maintained in serum-free keratinocyte growth medium (KGM-Gold; Lonza). For imaging purposes, BEAS-2B cells grown to 50% confluency were transduced with lentiviral vectors carrying *roGFP2* or *HyPer* genes targeting them to either the cytosol (roGFP2-cyto and HyPer-cyto) or mitochondria under the multiplicity of infection (MOI) of 5, as previously described (Tal et al. 2010). For catalase overexpression, BEAS-2B cells were transduced with an adenoviral vector encoding human catalase (AdCAT), green fluorescent protein (AdGFP), or empty vector for 4 hr using an MOI of 100. The adenoviral constructs were removed after transduction, and the cells were passaged in KGM-Gold.

*Cell exposure.* Growth factor-deprived BEAS-2B cells were exposed to DMSO control or 10–150 µM 1,2-NQ for 0–4 hr. Cells expressing *roGFP2* or *HyPer* were treated under observation with a Nikon Eclipse C1Si confocal imaging system (Nikon Instruments Inc., Melville, NY). In separate experiments, cells were analyzed using a PolarStar Optima microplate reader (BMG Labtech, Durham, NC) prior to and during treatment with 1,2-NQ. For gene expression analyses, BEAS-2B cells were exposed to 1–10 µM 1,2-NQ for 4 hr, and changes in the levels of specific transcripts were analyzed using real-time polymerase chain reaction (RT-PCR). In some experiments, cells were pretreated 30 min with DMSO control or the inhibitors diphenyleneiodonium (DPI; 25 µM), carbonyl cyanide 3-chlorophenylhydrazone (CCCP; 10 µM), rotenone (10 µM), sodium azide (NaN_3_; 2 mM), potassium cyanide (KCN; 10 µM), or cyclosporine A (CyA; 10 µM) before 1,2-NQ treatment.

*Measurement of redox potential and H_2_O_2_.* Confocal microscopy analyses were conducted using a C1Si system equipped with an Eclipse Ti microscope (Nikon). Green fluorescence was derived from excitations at 404 and 488 nm, and emission was detected using a band-pass filter of 525/50 nm (Chroma, Bellows Falls, VT). The results were calculated as ratios of the emissions excited by 488 nm and 404 nm lasers sequentially with a scanning frequency of 60 sec. The optical settings for the plate reader were similar to those used in the microscope, with excitation at 485/12 nm and 400/10 nm and emission at 520/30 nm (Chroma).

*RT-PCR.* Subconfluent BEAS-2B cells were exposed to varying concentrations of 1,2-NQ for 0–4 hr. Relative gene expression in BEAS-2B cells was quantified using the real-time PCR, ABI Prism 7500 Sequence Detection System (Applied Biosystems, Foster City, CA). Total RNA was isolated using an RNeasy kit (Qiagen, Valencia, CA) and reverse transcribed to generate cDNA using a High Capacity cDNA Reverse Transcription kit (Applied Biosystems). Oligonucleotide primer pairs and dual-labeled fluorescent probes for *IL-8*, *COX-2*, heme oxygenase-1 (*HO-1*), β*-actin*, and catalase were obtained from Applied Biosystems. The relative abundance of mRNA levels was determined using TaqMan Universal Master Mix (Applied Biosystems) and the 2^−ΔΔC_T_^ method (Livak and Schmittgen 2001). β*-Actin* mRNA was used to normalize levels of the mRNAs of interest.

*Statistical analysis.* Imaging data were collected with Nikon EZ-C1 software. An average of 5–10 cells was collected as regions of interests in each experiment, and data were quantified using Nikon Elements software (Nikon). Data are expressed as mean ± SE of three repeated experiments. The linear regression of plate reader results was calculated with GraphPad Prism (GraphPad Software, La Jolla, CA), and the slope of the regression line was plotted against 1,2-NQ concentrations. Pairwise comparisons were carried out using Student’s *t*-test, with *p* < 0.05 taken as statistically significant.

## Results

**Figure 1:**
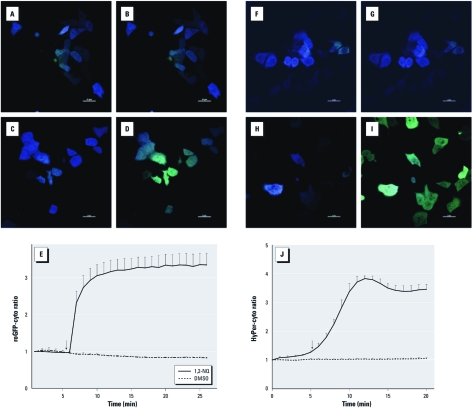
Measurement of redox change and H_2_O_2_ production visualized by roGFP-cyto (*A–E*) and HyPer-cyto (*F–J*) in BEAS-2B cells with and without 1,2‑NQ treatment. (*A–D*) BEAS-2B cells expressing roGFP-cyto were imaged under resting conditions (*A*, *C*) or after treatment with either DMSO (vehicle control; *B*) or 100 µM 1,2‑NQ (*D*); pseudocolor images correspond to a ratiometric calculation obtained by dividing fluorescence intensities acquired at 404 nm laser excitation over that obtained under 488 nm illumination. (*F–I*) Cells expressing HyPer-cyto were visualized before (*F*,*H*) and after treatment with DMSO (*G*) or 100 µM 1,2‑NQ (*I*). Pseudocolor images were generated from the ratio of 510 emission intensity under 488 nm over 404 nm excitations. In *A–D* and *F–I*, bars = 20 µm. (*E,J*) Time courses of redox changes monitored by roGFP-cyto ratios (*E*) and HyPer-cyto ratios (*J*) in cells stimulated with DMSO or 100 µM 1,2‑NQ. Arrows indicate the time DMSO or 100 µM 1,2‑NQ was added; values shown are mean ± SE (*n *= 3).

*1,2-NQ induces rapid oxidant changes.* We used the genetically encoded fluorescent probes roGFP-cyto and HyPer-cyto to monitor changes in redox potential and H_2_O_2_ production, respectively, in BEAS-2B cells exposed to 1,2-NQ. The cells were observed for 5 min to establish a baseline signal prior to treatment with either vehicle control or 100 µM 1,2-NQ for 15 min. As shown in Figure 1, treatment with 100 µM 1,2-NQ induced a rapid increase in the ratiometric fluorescence intensity of cytosolic roGFP2, corresponding to a marked loss of intracellular reducing potential that peaked and stabilized at 20 min (Figure 1C–E). The intracellular redox potential in cells exposed to vehicle alone remained stable during the same time period (Figure 1E). Similarly, cells expressing HyPer-cyto responded with an increase in fluorescence ratio intensity, indicating elevated levels of H_2_O_2_ after exposure to 1,2-NQ compared with control cells (Figure 1H–J).

**Figure 2:**
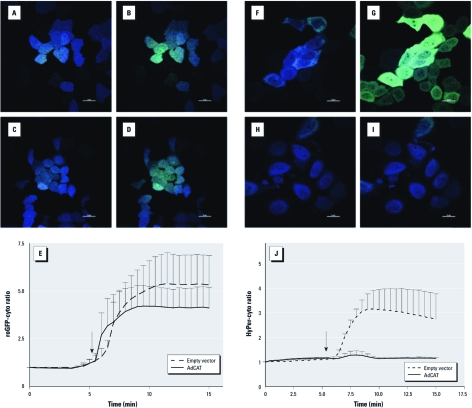
Catalase overexpression blunted 1,2‑NQ–induced hydrogen peroxide signals but not redox changes. Stably transduced BEAS-2B cells expressing roGFP-cyto (*A*–*D*) or HyPer-cyto (*F*–*I*) that received either an empty vector (*A*, *B*, *F*, *G*) or an adenoviral vector encoding catalase (AdCAT; *C*, *D*, *H*, *I*) were exposed to DMSO (*B,G*) or 100 µM 1,2‑NQ (*D,I*). In *A–D* and *F–I*, bars = 20 µm. (*E,J*) Time courses of roGFP-cyto ratios (*E*) or HyPer-cyto (*J*) were plotted for cells receiving empty vector or AdCAT. Arrows indicate the the time DMSO or 100 µM 1,2‑NQ was added; values shown are mean ± SE (*n *= 3).

*Overexpression of catalase blunts 1,2-NQ–induced H_2_O_2_ production.* To explore the interaction between changes in redox potential and H_2_O_2_ generation, we studied the effect of 1,2-NQ in BEAS-2B cells overexpressing catalase. Preliminary experiments established that catalase mRNA levels were 4 times higher in BEAS-2B cells transduced with AdCAT compared with controls (data not shown). Treatment of catalase-overexpressing BEAS-2B cells with 100 µM 1,2-NQ induced a loss of reducing potential that was not significantly different from that observed in BEAS-2B cells transduced with an empty vector (Figure 2A–E). In contrast, 1,2-NQ–induced H_2_O_2_ production was effectively ablated in BEAS-2B cells overexpressing catalase (Figure 2F–J).

**Figure 3:**
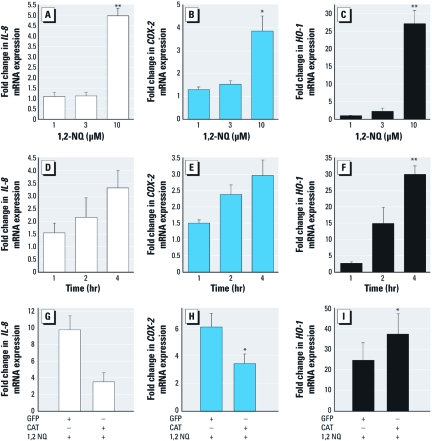
Dose- and time-dependent 1,2‑NQ–induced inflammatory and adaptive gene expression were differentially inhibited by catalase overexpression. Levels of *IL-8* (*A*,*D*,*G*), *COX‑2* (*B*,*E*,*H*), and *HO-1* (*C*,*F*,*I*) mRNA were measured using TaqMan-based RT-PCR, normalized to levels of β*-actin* mRNA, and expressed as fold increases over DMSO (vehicle control). For *A*–*C* and *G*–*I,* cells were exposed for 4 hr. For *D–F*, cells were treated with 10 µM 1,2‑NQ. (*G,H,I*) Transcript levels after treatment with 1,2‑NQ in BEAS-2B cells transduced with AdCAT or AdGFP. Values shown are mean ± SE; *n *= 3. *p < 0.05, and **p < 0.01.

*Overexpression of catalase differentially inhibits 1,2-NQ–induced gene expression.* We next examined the effect of 1,2-NQ exposure on the expression of the proinflammatory genes *IL-8* and *COX-2* and the adaptive, oxidant responsive gene *HO-1*. Exposure of BEAS-2B cells to 1–10 µM 1,2-NQ or vehicle for 0–4 hr resulted in dose- and time-dependent inductions in *IL-8*, *COX-2*, and *HO-1* mRNA (Figure 3A–F), with maximal respective increases of 5-, 4-, and 30-fold relative to vehicle controls observed at 4 hr of exposure. To test the mechanistic link between gene expression and oxidant responses, we determined the effect of 1,2-NQ exposure on the induction of *IL-8*, *COX-2*, and *HO-1* transcripts in BEAS-2B cells overexpressing catalase. Relative to control cells transduced with AdGFP, overexpression of catalase blunted the increases in *IL-8* and *COX-2* mRNA induced by treatment with 10 µM 1,2-NQ for 4 hr (Figure 3G,H). However, the induction of *HO-1* gene expression by 1,2-NQ was significantly augmented in BEAS-2B cells that overexpressed catalase (Figure 3I), indicating a differential role for H_2_O_2_ in 1,2-NQ–induced inflammatory and adaptive gene expression.

**Figure 4:**
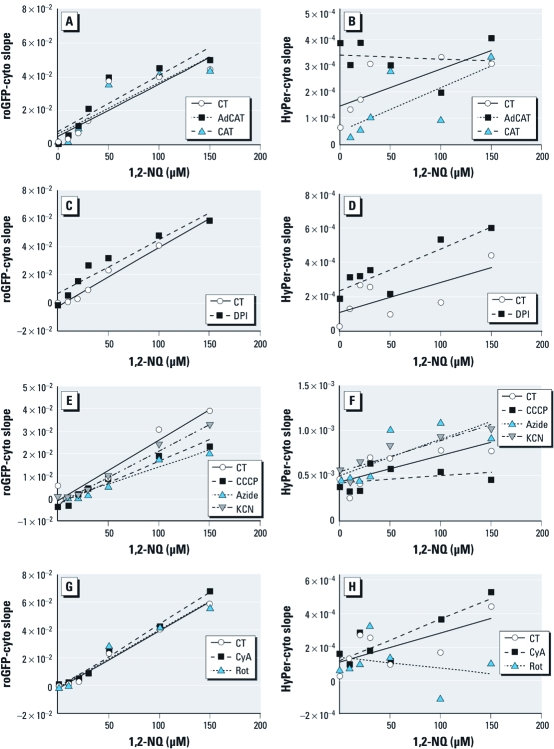
1,2‑NQ-induces mitochondrial H_2_O_2_ production. Redox potential and hydrogen peroxide levels were monitored in roGFP-cyto‑ (*A*,*C*,*E*,*G*) or HyPer-cyto‑ (*B*,*D*,*F*,*H*) expressing BEAS-2B cells exposed to 0–150 µM 1,2‑NQ. (*A*,*B*) Responses to 1,2 NQ in AdCAT BEAS-2B cells relative to wild-type BEAS-2B cells (control; CT), and in wild-type BEAS-2B cells exposed in the absence (CT) or presence of exogenous catalase (CAT). (*C–H*) BEAS-2B cells were pretreated with vehicle or the NADPH oxidase inhibitor DPI (25 µM; *C*, *D*), 10 µM of the mitochondrial inhibitors CCCP (*E,F*), KCN (*E,F*), CyA (*G,H*), or rotenone (Rot; *G,H*) or 2 mM azide (*E*,*F*). Values shown are mean slopes of linear regression analyses of fluorescence intensity (*n = *3), with error bars omitted for clarity.

*1,2-NQ induces intracellular production of H_2_O_2._* To identify the source of H_2_O_2_ production shown in Figure 1, we investigated possible mechanisms through which 1,2-NQ exposure of BEAS-2B cells could result in the generation of H_2_O_2_. To investigate the possibility that 1,2-NQ generates H_2_O_2_ extracellularly, we used a plate reader assay to monitor fluorescence changes in BEAS-2B cells expressing roGFP-cyto or HyPer-cyto with various 1,2-NQ concentrations (10–150 µM) in the presence or absence of exogenous catalase. As shown in Figure 4B, the inclusion of extracellular catalase did not significantly affect the magnitude or time of onset of 1,2-NQ–induced H_2_O_2_ generation in BEAS-2B cells, as detected by HyPer-cyto. However, in agreement with the microscopy findings shown in Figure 2, adenoviral-mediated overexpression of catalase in BEAS-2B cells ablated H_2_O_2_ production induced by 1,2-NQ treatment (Figure 4B). Neither extracellular catalase nor overexpression of catalase had any effect on the loss of cytoplasmic-reducing potential observed in roGFP-cyto expressing BEAS-2B cells treated with 1,2-NQ (Figure 4A).

*Identification of the mitochondrion as the source of 1,2-NQ–induced H_2_O_2_.* The data shown in Figure 4 indicated that 1,2-NQ exposure elevates the intracellular concentration of H_2_O_2_, suggesting the involvement of a cellular process. We therefore examined potential cellular sources of H_2_O_2_ generation in 1,2-NQ-treated cells. We first tested the involvement of H_2_O_2_ generation at the cell membrane by pretreating the cells with the specific NADPH oxidoreductase inhibitor DPI 30 min prior to the addition of 10–150 µM 1,2-NQ. We observed no significant differences in the production of H_2_O_2_ in cells exposed to 1,2-NQ in the presence of DPI relative to cells pretreated with vehicle alone (Figure 4D). We therefore turned our attention to possible mitochondrial sources of H_2_O_2_, using the mitochondrial inhibitors CCCP, NaN_3_, KCN, CyA, and rotenone. Of these inhibitors, CCCP (a mitochondrial membrane potential uncoupler) and rotenone (a mitochondrial complex I inhibitor) showed an effect on 1,2-NQ–induced H_2_O_2_ in BEAS-2B cells (Figure 4F,H). None of the inhibitors showed significant effects on 1,2-NQ–induced redox changes (Figure 4C,E,G). These findings implicated the mitochondrial respiratory chain as the source of 1,2-NQ–induced H_2_O_2_ production.

**Figure 5:**
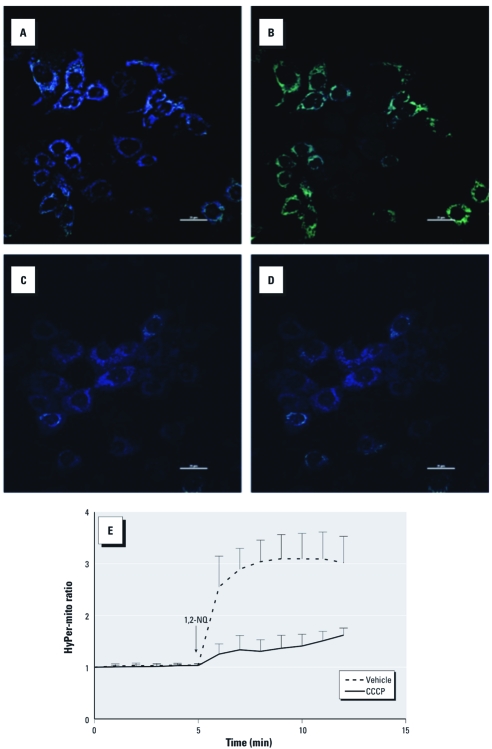
Confocal imaging of 1,2‑NQ–induced H_2_O_2_ production in mitochondria of BEAS-2B cells expressing HyPer-mito. Mitochondrial H_2_O_2_ was monitored as the ratio of HyPer-mito fluorescence emission intensity under 488/404 nm excitation in cells preincubated with DMSO vehicle (*A*,*B*) or 10 µM CCCP (*C*,*D*) before (*A*,*C*) and after (*B*, *D*) exposure to 100 µM 1,2‑NQ. In *A–D*, bars = 20 µm. (*E*) Plot of H_2_O_2_ production in BEAS-2B cells expressing HyPer-mito and pretreated with vehicle or 10 µM CCCP before the addition of 100 µM 1,2‑NQ (mean ± SE; *n* = 3).

We then examined BEAS-2B cells expressing Hyper-mito, a version of the H_2_O_2_ sensor that is targeted to the mitochondrial inner membrane. Exposure to 100 µM 1,2-NQ resulted in an elevation of ratiometric HyPer-mito fluorescence signal intensity, indicating elevated concentrations of H_2_O_2_ in the mitochondria (Figure 5A,B,E). 1,2-NQ–induced production of mitochondrial H_2_O_2_ was effectively suppressed by pretreatment of the cells with 10 µM CCCP (Figure 5C–E). These data showed 1,2-NQ–induced generation of H_2_O_2_ in the mitochondrion and further established mitochondrial respiration as the source of H_2_O_2_ production.

**Figure 6:**
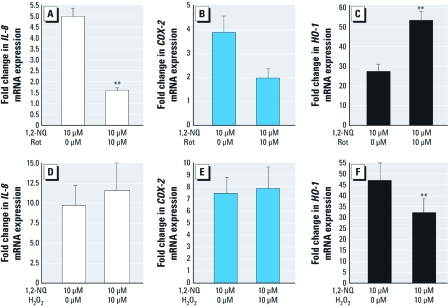
Differential role of mitochondrial H_2_O_2_ in 1,2‑NQ–induced gene expression in BEAS-2B cells pretreated with DMSO vehicle, the mitochondrial complex I inhibitor rotenone (Rot; 10 µM, 30 min), or H_2_O_2_ (30 µM, 10 sec) prior to the addition of 10 µM 1,2‑NQ for 4 hr. mRNA levels of *IL-8 *(*A*,*D*), *COX‑2*(*B*,*E*), and *HO-1 *(*C*,*F*) were measured using TaqMan-based RT-PCR, normalized to levels of β*-actin* mRNA, and expressed as fold increases over vehicle control (mean ± SE; *n* = 3). **p < 0.01.

*1,2-NQ–induced gene expression is differentially linked to mitochondrial activity and H_2_O_2_ availability.* We examined the role of mitochondrial metabolism in 1,2-NQ–induced inflammatory and adaptive gene expression by pretreating cells with rotenone for 30 min before exposure to 10 µM 1,2-NQ. Rotenone inhibited the induction of *IL-8* and *COX-2* expression by 1,2-NQ (Figure 6A,B). In marked contrast, the induction of *HO-1* mRNA by 1,2-NQ was potentiated by rotenone pretreatment (Figure 6C). This finding, combined with the earlier observation that catalase overexpression also enhanced the induction of *HO-1* mRNA by 1,2-NQ, led us to hypothesize that H_2_O_2_ limits 1,2-NQ–induced increases in *HO-1* mRNA. We therefore tested this hypothesis directly by adding 30 µM H_2_O_2_ immediately before 1,2-NQ treatment of BEAS-2B cells. As shown in Figure 6F, the addition of exogenous H_2_O_2_ significantly blunted the induction of *HO-1* expression by 1,2-NQ (Figure 6F). H_2_O_2_ pretreatment had no effect on 1,2-NQ–induced *IL-8* and *COX-2* expression (Figure 6D,E).

## Discussion

Oxidant effects are a commonly reported mechanistic feature of the toxicity of environmental agents. In the present study, we expanded our previously described integrated imaging approach to the investigation of mitochondrial dysfunction (Cheng et al. 2010) to include inflammatory and adaptive gene expression changes induced by an environmental electrophile capable of inducing multiple types of oxidant stress. This study presents a mechanistic link between early oxidant events resulting from exposure to 1,2-NQ and downstream toxicological effects, specifically alterations in the expression of genes involved in inflammatory and adaptive responses in a human bronchial epithelial cell line. In preliminary studies we observed similar oxidant responses and changes in gene expression in primary cultures of human airway epithelium (Cheng WY, unpublished data).

As one of the organic components of the ubiquitous air contaminant DEP (Cho et al. 2004; Inoue et al. 2007b; Jakober et al. 2007), 1,2-NQ has been shown to induce airway inflammation and to initiate deleterious effects through covalent modification or ROS generation (Inoue et al. 2007a, 2007b). Both activating and inhibitory effects of 1,2-NQ have been reported. For instance, Kikuno et al. (2006) reported that 1,2-NQ induces vanilloid receptor and epidermal growth factor receptor signaling, leading to guinea pig tracheal contraction. Inhibitory signaling effects associated with 1,2-NQ include impairment of cAMP response element–binding protein (CREB) (Endo et al. 2007) and lipopolysaccharide-induced nuclear factor kappa B (NFκB) DNA binding activities (Sumi et al. 2010). In addition, the cytoplasm, endoplasmic reticulum, nucleus, and mitochondrion are all major targets for 1,2-NQ–induced toxicity through protein modification in lung epithelial cells (Lame et al. 2003). Thus, the high reactivity of 1,2-NQ can result in a diversity of molecular effects that are likely dependent on concentration and also show cell type specificity.

In this study, we used the genetically encoded fluorescence reporters roGFP2 and HyPer to detect redox changes and H_2_O_2_ production, respectively. The exposure of 1,2-NQ induced rapid responses in both roGFP2 and HyPer in the cytosol of BEAS-2B cells, indicating an acute oxidative burden stimulated by this compound. The generation of ROS and changes in redox balance can be seen as related events. However, the observation that catalase overexpression blunted the 1,2-NQ–induced increase in H_2_O_2_ production without affecting the changes in redox potential suggests that H_2_O_2_ production is not the cause of the redox changes. Furthermore, overexpression of catalase also protected against 1,2-NQ–induced *IL-8* and *COX-2* expression, indicating that 1,2-NQ–stimulated H_2_O_2_ production is involved in the induction of inflammatory responses. This is in agreement with reports of the involvement of H_2_O_2_ in the activation of signaling pathways that regulate proinflammatory genes, such as *NF*κ*B*, *p38*, and *JNK* (Groeger et al. 2009). However, the addition of 30 µM H_2_O_2_ did not induce a statistically significant increase in *IL-8* or *COX-2* expression. This may reflect a requirement for H_2_O_2_ to act as a second messenger at specific subcellular compartments in order to initiate inflammatory gene expression.

An unexpected finding is that 1,2-NQ–induced *HO-1* expression in BEAS-2B cells was not mediated by H_2_O_2_. On the contrary, the magnitude of *HO-1* induction by 1,2-NQ was enhanced by removal of H_2_O_2_. Specifically, catalase expression and impairment of mitochondrial electron transport, which effectively decrease H_2_O_2_ concentrations and production, respectively, both potentiated 1,2-NQ–induced increases in *HO-1* mRNA. Furthermore, direct evidence for the suppressive effect of H_2_O_2_ on 1,2-NQ–induced *HO-1* expression was also obtained using exogenous H_2_O_2_. A similar finding was reported by Miura et al. (2011), who showed that pretreatment with catalase did not protect against 1,2-NQ–induced activation of nuclear factor (erythroid-derived 2)-like 2 (Nrf2), which is a regulator of *HO-1* gene expression. This is a seemingly paradoxical finding, as H_2_O_2_ is a known inducer of the Nrf2 pathway that regulates *HO-1* expression (Fourquet et al. 2010). One explanation for these observations may be that 1,2-NQ–induced *HO-1* expression requires electrophilic attack on a susceptible regulatory target, possibly a protein thiol, that is rendered unreactive to 1,2-NQ when oxidized by H_2_O_2_.

A parallel for H_2_O_2_-mediated inactivation of protein thiols is found in redox regulation of protein tyrosine phosphatases, in which the cysteine thiolate in the catalytic center of the enzyme is reversibly oxidized by H_2_O_2_ (Samet and Tal 2010). Using benzoquinone as the model toxicant, Mason and Liebler (2000) observed cysteine as a preferred target for quinone-induced toxicity. Recently, Miura et al. (2011) reported that Nrf2 activation by 1,2-NQ was mediated by covalent modification and subsequent degradation of Keap1. These studies point to cellular cysteine thiol groups as primary targets of electrophilic naphthoquinone attack by covalent modification (Lame et al. 2003). From this perspective, it is intriguing that 1,2-NQ has been shown to attack and inactivate the protein tyrosine phosphatase PTP1B, albeit at an allosteric site (Iwamoto et al. 2007). These observations lead us to speculate that biomolecular covalent modifications by 1,2-NQ are involved in *HO-1* gene expression induced by electrophilic attack. Detailed studies will be needed to elucidate the signaling mechanisms that underlie 1,2-NQ–induced gene expression.

A variety of metabolic processes are potential targets for xenobiotic-induced ROS production. Although quinone species that undergo redox cycling can generate ROS in cell-free aqueous environments (Le et al. 2007), the lack of an effect of extracellular catalase in suppressing the 1,2-NQ–induced HyPer signal excluded an extracellular redox process as a source of the H_2_O_2_. The presence of exogenous catalase would also be expected to scavenge H_2_O_2_ generated by membrane oxidoreductases because NADPH oxidases generate H_2_O_2_ in the extracellular space (Miller et al. 2010). The failure of the oxidoreductase activity inhibitor DPI to suppress HyPer signals is consistent with this notion and thus helped shift the focus to the mitochondria as a source of 1,2-NQ–induced H_2_O_2_ in this study.

The observation of H_2_O_2_-dependent fluorescence in the mitochondria confirmed that the mitochondrion is the site of H_2_O_2_ production in BEAS-2B cells exposed to 1,2-NQ. Of the variety of mitochondrial inhibitors used in this study that target membrane potential (CCCP), complex I (rotenone), complex IV (KCN and NaN_3_), and the permeability transition pore (CyA), only CCCP and rotenone blunted 1,2-NQ–induced HyPer signals, indicating that the molecular target for 1,2-NQ–stimulated H_2_O_2_ is associated with components of the upstream mitochondrial respiratory chain. A similar mitochondrial dysfunction was observed by Xia et al. (2004) who exposed a mouse macrophage cell line to a quinone-enriched polar fraction of DEP. Furthermore, in the present study, pretreatment with rotenone diminished 1,2-NQ–induced *IL-8* and *COX-2* gene expression, further establishing the functional link between the formation of mitochondrial H_2_O_2_ and inflammatory gene expression.

The ambient concentration of 1,2-NQ has been reported to range from 13 to 53 µg/g DEP (Cho et al. 2004; Valavanidis et al. 2006). Given the ubiquitous nature of DEP as a constituent of ambient particulate matter (PM), plausible real-world scenarios may result in exposure of airway epithelial cells to deposited doses of 1,2-NQ during a 3-hr inhalational exposure that are about 10-fold lower than those used in this study [see Supplemental Material (http://dx.doi.org/10.1289/ehp.1104055) for supporting calculations and assumptions]. Moreover, 1,2-NQ is representative of a class of organic constituents of ambient PM that includes other quinones as well as polyaromatic hydrocarbons that may be metabolized to redox active quinones (Cho et al. 2004; Valavanidis et al. 2006).

**Figure 7:**
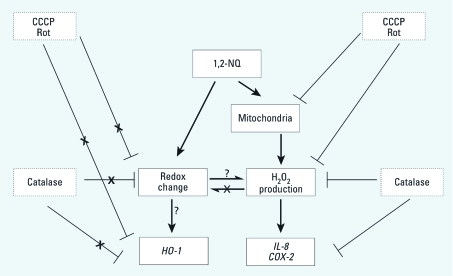
Proposed scheme of 1,2‑NQ–induced effects.

Most studies on environmental electrophiles such as 1,2-NQ have focused on the highly reactive electrophilic properties of these compounds. Here we were able to measure early oxidative events in real time and correlate them mechanistically to gene expression changes associated with adverse responses to electrophilic exposure. In this study, we demonstrate that 1,2-NQ induces mitochondrial H_2_O_2_ production that leads to inflammatory gene expression but not the accompanying loss of reducing potential observed in the cytosol. 1,2-NQ also induces *HO-1* expression; however, our data show that it does so through a mechanism that is actually opposed by the availability of H_2_O_2_. Thus, these findings reveal dissociation between H_2_O_2_ production and the loss of reducing potential induced by a frank electrophile (Figure 7). Ascertaining whether the loss of reducing potential is a consequence or a cause in the induction of HO-1 by 1,2-NQ requires further investigation. Taken as a whole, our experimental strategy in this study represents an integrated approach for the systematic study of oxidative events that underlie adverse cellular responses to xenobiotic exposure. From a public health perspective, the inflammatory and adaptive responses induced by 1,2-NQ are consistent with the inflammatory and immunotoxic effects that are associated with human exposure to DEP and ambient PM.

## Supplemental Material

(86 KB) PDF

## References

[r1] Bai Y, Suzuki AK, Sagai M (2001). The cytotoxic effects of diesel exhaust particles on human pulmonary artery endothelial cells in vitro: role of active oxygen species.. Free Radic Biol Med.

[r2] Becker S, Mundandhara S, Devlin RB, Madden M (2005). Regulation of cytokine production in human alveolar macrophages and airway epithelial cells in response to ambient air pollution particles: further mechanistic studies.. Toxicol Appl Pharmacol.

[r3] Cheng WY, Tong H, Miller EW, Chang CJ, Remington J, Zucker RM (2010). An integrated imaging approach to the study of oxidative stress generation by mitochondrial dysfunction in living cells.. Environ Health Perspect.

[r4] Cho AK, Stefano ED, You Y, Rodriguez CE, Schmitz DA, Kumagai Y (2004). Determination of four quinones in diesel exhaust particles, SRM 1649a, and atmospheric PM_2.5_.. Aerosol Sci Technol.

[r5] Endo A, Sumi D, Kumagai Y. (2007). 1,2-Naphthoquinone disrupts the function of cAMP response element-binding protein through covalent modification.. Biochem Biophys Res Commun.

[r6] Ercal N, Gurer-Orhan H, Aykin-Burns N. (2001). Toxic metals and oxidative stress part I: mechanisms involved in metal-induced oxidative damage.. Curr Top Med Chem.

[r7] Fourquet S, Guerois R, Biard D, Toledano MB (2010). Activation of NRF2 by nitrosative agents and H_2_O_2_ involves KEAP1 disulfide formation.. J Biol Chem.

[r8] Groeger G, Quiney C, Cotter TG (2009). Hydrogen peroxide as a cell-survival signaling molecule.. Antioxid Redox Signal.

[r9] Hanson GT, Aggeler R, Oglesbee D, Cannon M, Capaldi RA, Tsien RY (2004). Investigating mitochondrial redox potential with redox-sensitive green fluorescent protein indicators.. J Biol Chem.

[r10] Inoue K, Takano H, Hiyoshi K, Ichinose T, Sadakane K, Yanagisawa R (2007a). Naphthoquinone enhances antigen-related airway inflammation in mice.. Eur Respir J.

[r11] Inoue K, Takano H, Ichinose T, Tomura S, Yanagisawa R, Sakurai M (2007b). Effects of naphthoquinone on airway responsiveness in the presence or absence of antigen in mice.. Arch Toxicol.

[r12] Iwamoto N, Sumi D, Ishii T, Uchida K, Cho AK, Froines JR (2007). Chemical knockdown of protein-tyrosine phosphatase 1B by 1,2-naphthoquinone through covalent modification causes persistent transactivation of epidermal growth factor receptor.. J Biol Chem.

[r13] Jakober CA, Riddle SG, Robert MA, Destaillats H, Charles MJ, Green PG (2007). Quinone emissions from gasoline and diesel motor vehicles.. Environ Sci Technol.

[r14] Kelly KA, Havrilla CM, Brady TC, Abramo KH, Levin ED (1998). Oxidative stress in toxicology: established mammalian and emerging piscine model systems.. Environ Health Perspect.

[r15] Kikuno S, Taguchi K, Iwamoto N, Yamano S, Cho AK, Froines JR (2006). 1,2-Naphthoquinone activates vanilloid receptor 1 through increased protein tyrosine phosphorylation, leading to contraction of guinea pig trachea.. Toxicol Appl Pharmacol.

[r16] Kumagai Y, Arimoto T, Shinyashiki M, Shimojo N, Nakai Y, Yoshikawa T (1997). Generation of reactive oxygen species during interaction of diesel exhaust particle components with NADPH-cytochrome P450 reductase and involvement of the bioactivation in the DNA damage.. Free Radic Biol Med.

[r17] Kuroda H, Takeno M, Murakami S, Miyazawa N, Kaneko T, Ishigatsubo Y. (2010). Inhibition of heme oxygenase-1 with an epidermal growth factor receptor inhibitor and cisplatin decreases proliferation of lung cancer A549 cells.. Lung Cancer.

[r18] Kuwahara I, Lillehoj EP, Lu W, Singh IS, Isohama Y, Miyata T (2006). Neutrophil elastase induces *IL-8* gene transcription and protein release through p38/NF-κB activation via EGFR transactivation in a lung epithelial cell line.. Am J Physiol Lung Cell Mol Physiol.

[r19] Lame MW, Jones AD, Wilson DW, Segall HJ (2003). Protein targets of 1,4-benzoquinone and 1,4-naphthoquinone in human bronchial epithelial cells.. Proteomics.

[r20] Le SB, Hailer MK, Buhrow S, Wang Q, Flatten K, Pediaditakis P (2007). Inhibition of mitochondrial respiration as a source of adaphostin-induced reactive oxygen species and cytotoxicity.. J Biol Chem.

[r21] Li N, Sioutas C, Cho A, Schmitz D, Misra C, Sempf J (2003). Ultrafine particulate pollutants induce oxidative stress and mitochondrial damage.. Environ Health Perspect.

[r22] Livak KJ, Schmittgen TD (2001). Analysis of relative gene expression data using real-time quantitative PCR and the 2^–ΔΔC_T_^ method.. Methods.

[r23] Mason DE, Liebler DC (2000). Characterization of benzoquinone-peptide adducts by electrospray mass spectrometry.. Chem Res Toxicol.

[r24] Miller EW, Dickinson BC, Chang CJ (2010). Aquaporin-3 mediates hydrogen peroxide uptake to regulate downstream intracellular signaling.. Proc Natl Acad Sci USA.

[r25] Miura T, Shinkai Y, Jiang HY, Iwamoto N, Sumi D, Taguchi K (2011). Initial response and cellular protection through the Keap1/Nrf2 system during the exposure of primary mouse hepatocytes to 1,2-naphthoquinone.. Chem Res Toxicol.

[r26] Monks TJ, Hanzlik RP, Cohen GM, Ross D, Graham DG (1992). Quinone chemistry and toxicity.. Toxicol Appl Pharmacol.

[r27] Rahman I, MacNee W. (2000). Oxidative stress and regulation of glutathione in lung inflammation.. Eur Respir J.

[r28] Reddel RR, Ke Y, Gerwin BI, McMenamin MG, Lechner JF, Su RT (1988). Transformation of human bronchial epithelial cells by infection with SV40 or adenovirus-12 SV40 hybrid virus, or transfection via strontium phosphate coprecipitation with a plasmid containing SV40 early region genes.. Cancer Res.

[r29] Rodriguez CE, Shinyashiki M, Froines J, Yu RC, Fukuto JM, Cho AK (2004). An examination of quinone toxicity using the yeast *Saccharomyces cerevisiae* model system.. Toxicology.

[r30] Samet JM, Tal TL (2010). Toxicological disruption of signaling homeostasis: tyrosine phosphatases as targets.. Annu Rev Pharmacol Toxicol.

[r31] Santa-Maria I, Smith MA, Perry G, Hernéndez F, Avila J, Moreno FJ (1740). 2005. Effect of quinones on microtubule polymerization: a link between oxidative stress and cytoskeletal alterations in Alzheimer’s disease.. Biochim Biophys Acta.

[r32] Simmons SO, Fan C-Y, Yeoman K, Wakefield J, Ramabhadran R (2011). NRF2 oxidative stress induced by heavy metals is cell type dependent.. Curr Chem Genomics.

[r33] Steinberg JJ, Gleeson JL, Gil D (1990). The pathobiology of ozone-induced damage.. Arch Environ Health.

[r34] Sumi D, Akimori M, Inoue K, Takano H, Kumagai Y. (2010). 1,2-Naphthoquinone suppresses lipopolysaccharide-dependent activation of IKKβ/NF-κB/NO signaling: an alternative mechanism for the disturbance of inducible NO synthase-catalyzed NO formation.. J Toxicol Sci.

[r35] Sun Y, Taguchi K, Sumi D, Yamano S, Kumagai Y. (2006). Inhibition of endothelial nitric oxide synthase activity and suppression of endothelium-dependent vasorelaxation by 1,2-naphthoquinone, a component of diesel exhaust particles.. Arch Toxicol.

[r36] Tal TL, Simmons SO, Silbajoris R, Dailey L, Cho SH, Ramabhadran R (2010). Differential transcriptional regulation of IL-8 expression by human airway epithelial cells exposed to diesel exhaust particles.. Toxicol Appl Pharmacol.

[r37] Tsatsanis C, Androulidaki A, Venihaki M, Margioris AN (2006). Signalling networks regulating cyclooxygenase-2.. Int J Biochem Cell Biol.

[r38] Valavanidis A, Fiotakis K, Vlahogianni T, Papadimitrious V, Pantikaki V. (2006). Determination of selective quinones and quinoid radicals in airborne particulate matter and vehicular exhaust particles.. Environ Chem.

[r39] Valko M, Morris H, Cronin MT (2005). Metals, toxicity and oxidative stress.. Curr Med Chem.

[r40] Xia T, Korge P, Weiss JN, Li N, Venkatesen MI, Sioutas C (2004). Quinones and aromatic chemical compounds in particulate matter induce mitochondrial dysfunction: implications for ultrafine particle toxicity.. Environ Health Perspect.

