# Supplementary material for: Linking Oxidative Events to Inflammatory and Adaptive Gene Expression Induced by Exposure to an Organic Particulate Matter Component
Source: Environ Health Perspect. 2011 Oct 13;120(2):267–74. doi: 10.1289/ehp.1104055 (PMC3279454; doi:10.1289/ehp.1104055)
Supplement: (86 KB) PDF [file ehp.1104055.s001.pdf]

SUPPLEMENTAL MATERIAL

Linking Oxidative Events to Inflammatory and Adaptive Gene Expression Induced by Exposure to an Organic PM Component

Wan-Yun Cheng[1]; Jenna Currier[2]; Philip A. Bromberg[3]; Robert Silbajoris[4]; Steven O. Simmons[5]; and James M. Samet[4]

[1]Department of Environmental Sciences and Engineering and [2]Curriculum in Toxicology, University of North Carolina, Chapel Hill, NC; [3] Center for Environmental Medicine, Asthma and Lung Biology, University of North Carolina at Chapel Hill, Chapel Hill; [4]Environmental Public Health Division, NHEERL, U.S. EPA, Chapel Hill, NC; [5]Integrated Systems Toxicology, National Health and Environmental Effects Research Laboratory, U.S. EPA, USA

Corresponding author:

James M. Samet, PhD

104 Mason Farm Rd.

EPA Human Studies Facility

Chapel Hill, NC 27599-7310

Phone: 1-919-966-0665

Fax: 1-919-962-6271

Email: Samet.James@EPA.gov

The concentration of 1,2-NQ in samples of DEP has been reported to be between 13.7 ± 3.0 and 53 ± 5 ug/g, which is equivalent to 86.6-335 nmols 1,2-NQ/g DEP (Cho et al. 2004; Valavanidis et al. 2006).

In certain settings such as bus depots, garages and tunnels concentrations of DEP can reach or exceed 300 ug/m^3 DEP (NTP 2011). Assuming a ventilatory rate of 1 m^3/hr , a 3 hr exposure would produce an inhalational exposure to 0.9 mg of DEP. Using the concentrations above, this translates into an inhalational exposure to 77.9-302 pmols of 1,2-NQ.

Using the Weibel model of dichotomously branched conducting airways for generation 8 (with the trachea representing generation 1) yields approximately 100 cm^2 of ciliated epithelium. Considering generations 8 thru 11, the area would be approximately 1000 cm^2 (Weibel 1963). Estimating an average depth of periciliary fluid of 5 microns, gives a total volume of between 50 and 500 ul. Under this scenario, human airway epithelial cells could be exposed to concentrations to concentrations of 1,2-NQ between 0.16 and 6 uM *in vivo*.

These calculations assume 100 % tracheobronchial deposition of the inhaled DEP in generations 8-11. In reality, the rate of deposition of inhaled DEP particles (0.1-0.5 um) is fairly uniform throughout the conducting airways and is expected to be approximately 5 % (Choi and Kim 2007). At this rate, and factoring in a total periciliary volume of 2.4 ml (corresponding to generations 1 thru 17) would result in an effective concentration experienced by airway epithelial cells *in vivo* of approximately 6 nM.

These scenarios ignore other factors such as clearance and dissolution rates of 1,2-NQ. On the other hand, these calculations do not take into account zonal accumulation at bifurcations and focal deposition in disease states such as COPD, which can elevate local concentrations significantly above those in a healthy lung . For instance, local particle deposition at carinal ridges involves a surface area that is only 100 um x 100 um which provides an enhancement factor up to 100-fold. Considering an area 3 mm x 3 mm (which is still small by comparison to the cell culture surface area used in this study) would yield an enhancement factor of approximately 7-fold (Balashazy et al, 2003).

References list

NTP. Diesel Exhaust Particulates. 2011. In: Report on Carcinogens, NTP 12th Report on Carcinogens, 153-156.

Cho AK, Stefano ED, You Y, Rodriguez CE, Schmitz DA, Kumagai Y, et al. 2004. Determination of four quinones in diesel exhaust particles, SRM 1649a, and atmospheric PM2.5. Aerosol Science and Tchnology 38(S1): 68-81.

Valavanidis A, Fiotakis K, Vlahogianni T, Papadimitrious V, Pantikaki V. 2006. Determination of Selective Quinones and Quinoid Radicals in Airborne Particulate Matter and Vehicular Exhaust Particles. Environmental Chemistry 3(2): 118-123.

Weibel ER. 1963. Morphometry of the Human Lung. New York: Springer Verlag and Academic Press, Berlin.

Choi JI, Kim CS. 2007. Mathematical analysis of particle deposition in human lungs: an improved single path transport model. Inhal Toxicol 19(11): 925-939.

Balashazy I, Hofmann W, Heistracher T. 2003. Local particle deposition patterns may play a key role in the development of lung cancer. J Appl Physiol 94(5): 1719-1725.
